# *Hsa-miR-335* regulates cardiac mesoderm and progenitor cell differentiation

**DOI:** 10.1186/s13287-019-1249-2

**Published:** 2019-06-27

**Authors:** Maryam Kay, Bahram Mohammad Soltani, Fahimeh Hosseini Aghdaei, Hassan Ansari, Hossein Baharvand

**Affiliations:** 1 Department of Genetics, Faculty of Biological Sciences, Tarbiat Modares University, Tehran, Iran; 2 Department of Stem Cells and Developmental Biology, Cell Science Research Center, Royan Institute for Stem Cell Biology and Technology, ACECR, Tehran, Iran; 3 Department of Developmental Biology, University of Science and Culture, Tehran, Iran; 4 Department of Molecular Genetics, Faculty of Biological Sciences, Tarbiat Modares University, 14115-111, Tehran, Iran; 5 Royan Institute, P.O. Box: 16635-148, Banihashem Sq., Banihashem St., Ressalat Highway, Tehran, 1665659911 Iran

**Keywords:** miRNA, Cardiomyocyte differentiation, hESC, WNT, TGFβ

## Abstract

**Background:**

WNT and TGFβ signaling pathways play critical regulatory roles in cardiomyocyte fate determination and differentiation. MiRNAs are also known to regulate different biological processes and signaling pathways. Here, we intended to find candidate miRNAs that are involved in cardiac differentiation through regulation of WNT and TGFβ signaling pathways.

**Methods:**

Bioinformatics analysis suggested *hsa-miR-335-3p* and *hsa-miR-335-5p* as regulators of cardiac differentiation. Then, RT-qPCR, dual luciferase, TOP/FOP flash, and western blot analyses were done to confirm the hypothesis.

**Results:**

Human embryonic stem cells (hESCs) were differentiated into beating cardiomyocytes, and these miRNAs showed significant expression during the differentiation process. Gain and loss of function of *miR-335-3p* and *miR-335-5p* resulted in *BRACHYURY*, *GATA4*, and *NKX2-5* (cardiac differentiation markers) expression alteration during the course of hESC cardiac differentiation. The overexpression of *miR-335-3p* and *miR-335-5p* also led to upregulation of *CNX43* and *TNNT2* expression, respectively. Our results suggest that this might be mediated through enhancement of WNT and TGFβ signaling pathways.

**Conclusion:**

Overall, we show that *miR-335-3p/5p* upregulates cardiac mesoderm (*BRACHYURY*) and cardiac progenitor cell (*GATA4* and *NKX2-5*) markers, which are potentially mediated through activation of WNT and TGFβ signaling pathways. Our findings suggest *miR-335-3p/5p* to be considered as a regulator of the cardiac differentiation process.

**Electronic supplementary material:**

The online version of this article (10.1186/s13287-019-1249-2) contains supplementary material, which is available to authorized users.

## Introduction

Human embryonic stem cells (hESCs) are pluripotent cells derived from the inner cell mass of blastocysts. These cells have self-renewal capacity and are able to differentiate into all derivatives of three germ layers [1]. The first lineage decision is chosen following gastrulation in which cells differentiate into the mesoderm, endoderm, and ectoderm [2]. Differentiation of hESCs is an ideal model for cardiogenesis research as these cells mimic the behavior of cells at early stages of embryonic development [3, 4]. Proper differentiation occurs by precise regulation of different transcription networks, signaling pathways, and epigenetic modifications [5, 6]. WNT/β catenin and TGFβ signaling pathways are known as two main and critical signaling pathways that regulate different stages of cardiac differentiation including mesendoderm cell commitment, cardiac progenitor differentiation, and final maturation [7–10]. Numerous factors tightly orchestrate these signaling pathways during differentiation [11]. MicroRNAs (miRNAs) are small (22 to 25 nucleotide in length) non-coding RNAs [12] that regulate gene expression and play important roles in proliferation, differentiation, cell fate decision, and various physiological processes [13–15]. Accumulating evidence has shown that miRNAs regulate signaling pathways related to cardiac differentiation at posttranscriptional and posttranslational levels [16, 17]. For instance, *miR-15*, *miR-16* [18], and *miR-430* [19] are identified as regulators of cell fate acquisition through targeting the TGFβ signaling pathway. *miR-1* and *miR-133* promote mesoderm formation [20] and *miR-499* promotes CPC differentiation into cardiomyocytes [21]. Here, we screen miRNAs that might be involved in cardiac differentiation through regulation of WNT/β catenin and TGFβ signaling pathways. Bioinformatics analyses indicate that *miR-335-3p* and *miR-335*-*5p* might regulate these two signaling pathways through targeting core members of the pathways. Gain- and loss-of-function studies were performed to verify the exact role of these two miRNAs in cardiac differentiation. Our findings demonstrate that these two miRNAs might regulate cardiac differentiation by activating WNT and TGFβ signaling pathways. This activation led to enhanced mesoderm cell commitment and promoted cardiac progenitor cell differentiation.

## Materials and methods

### Cell culture and differentiation

HEK293 and SW480 cells were maintained in Dulbecco’s modified Eagle’s medium (DMEM) (Gibco), supplemented with 10% heat-inactivated fetal bovine serum and 1% antibiotics (100 U/mL penicillin and 100 μg/mL streptomycin) (Gibco). Cells were grown at 37 °C in a humidified atmosphere with 5% CO_2_. The hESC line RH5 [22] was expanded under feeder-free conditions on Matrigel-coated plates. Cardiomyocyte differentiation occurred in a chemically defined medium, as previously described [23, 24] with minor modifications. Cells were stimulated with 20 ng/mL fibroblast growth factor 2 (FGF2), 20 ng/mL activin A, and 10 ng/mL BMP4 in the first 36 h for mesoderm induction; then, cells were treated with 20 ng/mL FGF2, 50 ng/mL BMP4, 0.5 mM retinoic acid, and 5 mM WNT inhibitor (IWP2) from day 1.5 to day 5. Finally, cells were treated with 5 ng/mL FGF2 and 10 ng/mL BMP4 which resulted in cardiomyocyte differentiation. Samples were collected at different time points (0, 0.5, 1, 1.5, 2, 5, and 12 days) of differentiation for expression analysis.

### Transfection of hESCs

Gain- and loss-of-function studies were done in (day 0) D0 of differentiation. The miRCURY LNA™ microRNA mimic (Exiqon, Denmark) for *miR-335-3p* (MIMAT0004703), *miR-335-5p* (MIMAT0000765), and mimic control as well as miRIDIAN microRNA *miR-335-3p* and *miR-335-5p* hairpin inhibitors and miRIDIAN microRNA hairpin inhibitor control (Dharmacon) were used for gain- and loss-of-function studies in which 8 × 10^5^ cells were plated in each 3.5-cm tissue culture dish, 24 h before transfection. When cells reached 80% confluence, they were transfected by 50 nM siRNA or 5 nM mimic structures using Lipofectamine® 3000 reagent, based on the manufacturer’s instructions. The efficiency of siRNAs and microRNA mimics transfection was evaluated using BLOCK-iT Alexa Fluor Red fluorescent oligo (Invitrogen).

### RNA extraction and quantitative RT-PCR

Total RNA of harvested cells was extracted using TRIzol™ reagent (Invitrogen, USA) according to the manufacturer’s protocol. The total RNA was used for cDNA synthesis after being treated with RNase-free DNase (Takara, Japan) in order to remove any DNA contamination. cDNAs were synthesized using RevertAid™ Reverse Transcriptase (Fermentase, Lithuania) according to the manufacturer’s instructions. For miRNA detection, polyA tail was added to 3′ end of RNAs before cDNA synthesis. RT-qPCR was performed using specific primers (Table S1) by StepOne Real-Time PCR system (Applied Biosystems). *GAPDH* and small nucleolar RNA, C/D box 48 (*SNORD48*) were used as internal controls for normalization of mRNAs and miRNA expression.

### Immunocytochemistry (ICC)

The seeded cells were washed once with phosphate buffered saline (PBS) and fixed with 4% (*w*/*v*) paraformaldehyde (PFA) for 15 min at room temperature. The cells were permeabilized using PBS containing 0.2% Triton X-100 for 10 min followed by blocking in a solution containing PBS and 10% Donkey serum for 1 h at room temperature. Next, cells were treated with primary antibody overnight at 4 °C. After three washes in PBS, cells were incubated with secondary antibody for 1 h at room temperature in the dark. Finally, cells were incubated with DAPI for nucleic acid staining and imaged with a fluorescent microscope (IX71, Olympus, Japan). Image overlays and contrast enhancement were performed using ImageJ software.

### Dual luciferase assay

Dual luciferase assay was utilized to validate the direct interaction between *miR-335-3p* and *miR-335-5p* and their target genes 3′-UTRs. To this aim, HEK293 cells were co-transfected with a psiCHECK-2 vectors including 3′-UTR of *APC*, *AXIN-I*, and *SMAD7* and *miR-335-3p*, *miR-335-5p* mimics, and siRNA structures in 48-well plates. As negative controls, mimic and siRNA scramble were used in co-transfection. Luciferase activity was measured 48 h after transfection using the Dual Luciferase Reporter Assay System (Promega, USA) according to the manufacturer’s instructions.

### TOP/FOP reporter assays

TOP/FOP reporter assays were carried out using the Dual-Glo luciferase assay kit (Promega), based on the manufacturer’s instructions. SW480 cells were transfected with 1μg of constitutively active vector encoding Renilla luciferase, responsive firefly luciferase reporter plasmid Top Flash, each mimic and siRNA corresponding to the *miR-335-3p*, *miR-335-5p* and their related scrambled. Cells were harvested after 48 h, and both firefly and Renilla luciferase activity were measured in three biological replicates according to the manufacturer’s instructions. The firefly luciferase activity was normalized against Renilla luciferase activity.

### Western blotting

The protein was extracted from the samples. Samples containing 40 μg purified protein were separated by 12% SDS/PAGE, transferred to PVDF membranes (Santa Cruz), and run at 100 V for 1.5 h at room temperature. The PVDF membrane was subsequently blocked by 5% BSA in phosphate-buffered saline (PBS) containing 0.1% Tween for 1 h at room temperature, followed by overnight incubation at 4 °C with phospho BRACHYURY primary antibodies (1:500, Cell Signaling). The blot membrane was washed and incubated with anti-rabbit secondary antibody (1:1000, Santa Cruz) for 1 h at room temperature. The protein bands were visualized by an enhanced chemiluminescence (ECL) detection system (Amersham, Piscataway, NJ). The membranes were stripped and re-probed with β-actin for verification of protein loading. The bands were quantified using an image analyzer program (ImageJ).

## Results

### Bioinformatics analyses introduced *miR-335* as a potential regulator of cardiac differentiation

WNT and TGFβ signaling pathways are known as main players in cardiac differentiation. In this study, we aimed to find out miRNAs that are crucially important for driving cardiac differentiation through regulation of WNT and TGFβ signaling pathways. To this aim, a four-step filtering approach was performed to nominate some miRNAs (Fig. 2a). At the first step, 973 miRNAs were predicted to target WNT and TGFβ signaling pathways using miRWalk, a target prediction resource that uses several miRNA target prediction tools. Then, considering the number of genes which are targeted by a miRNA in each pathway, the numbers of MREs (miRNA recognition elements) in 3′UTR sequences of each target gene, and the annealing and conservation status of each MRE, seven candidate miRNAs were chosen for further analyses (Additional file 1: Table S2). Among candidate miRNAs, *miR-335-3p/5p* hosted by *mesoderm-specific transcript (MEST)* gene were chosen for further investigations concerning its role in cardiogenesis.

### Differentiation of hESCs into cardiomyocytes

**Fig. 1:**
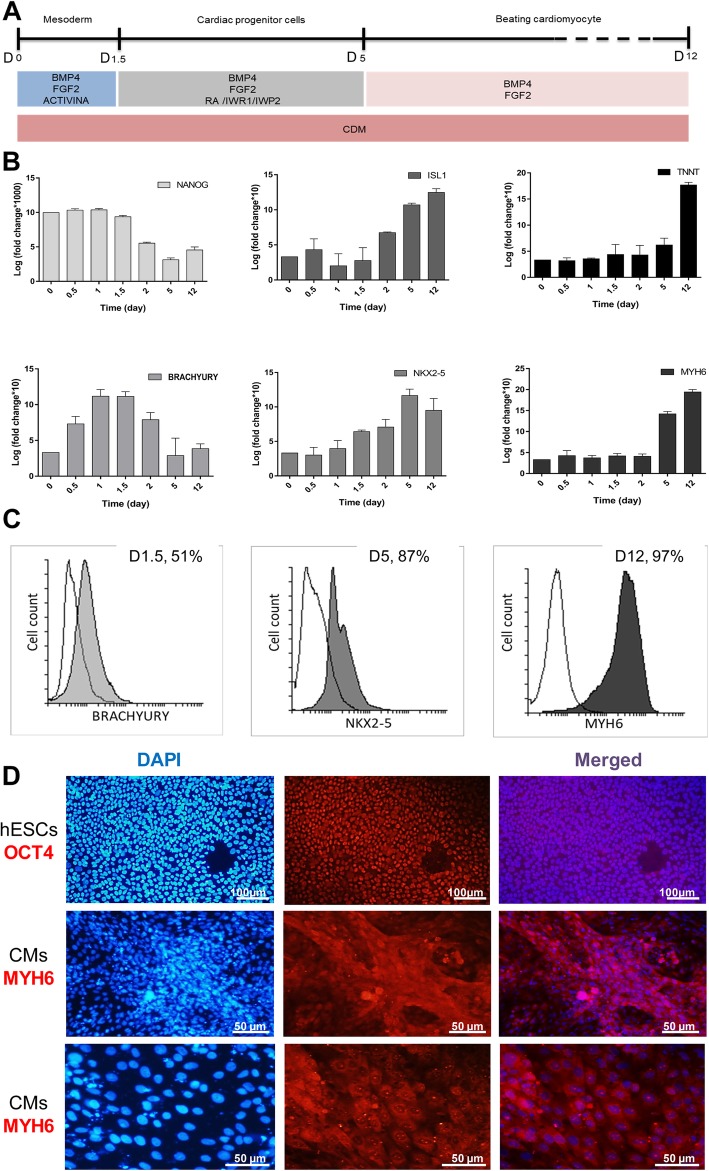
Characterization of hESC-derived cardiomyocyte. **a** Schematic description of cardiomyocyte differentiation protocol. Activin A and BMP4 compounds were used to induce differentiation of hESCs into mesoderm. Then, WNT inhibitor reagent (IWP2) was used to induce cardiac progenitor cell development followed by cardiomyocyte formation. **b** Time-dependent expression of *NANOG* (pluripotency marker), *BRACHYURY* (mesoderm marker), *NKX2-5*, *ISL1* (cardiac progenitor cell markers), *TNNT2*, and *MYH6* (cardiomyocyte markers) during the cardiac differentiation process. RT-qPCR data are presented as mean ± SEM normalized against day 0 data, for *n* = 3 independent experiments. *GAPDH* was used as a housekeeping gene. **c** Flow cytometry results confirmed the expression of *BRACHYURY* (day 1.5), *NKX2-5* (day 5), and *MYH6* (day 12) during the differentiation process. **d** Immunocytochemistry analysis of *OCT4* (pluripotent hESCs) and *MYH6* (cardiomyocytes) revealed the stemness potency of hESCs and successful differentiation of cardiomyocyte. Differentiated cardiomyocytes are shown in two magnitudes

In vitro differentiation of hESC, RH5, to cardiomyocyte-like cells was successfully performed (Fig. 1a). Different time points representing different cell fates during cardiac differentiation were considered (i.e., pluripotent stem cells (day 0), mesendoderm (day 1.5), cardiac progenitor (day 5), and cardiomyocyte cells (day 12)). *NANOG* stem cell marker expression was substantially decreased while the *BRACHYURY* (mesendodermal marker) expression level reached its highest level in mesendoderm stage (day 1.5) (Fig. 1b). In addition, *NKX2-5* and *ISL1* (early cardiac differentiation markers) expression levels elevated from day 2 and substantially increased during the process. As expected, cardiac-specific markers (*TNNT* and *MYH6*) were significantly increased at the final stage (day 12) (Fig. 1b), followed by spontaneous beating of the cardiac cells (Additional file 2: Movie S1). The flow cytometry analysis indicated that ~ 51% of the cells were *BRACHYURY* positive on day 1.5, ~ 87% of the cells were *NKX2-5* positive on day 5 (cardiac progenitor cells) and ~ 96% of the cells were *MYH6* positive on day 12 (the final step of cardiomyocyte differentiation) (Fig. 1c). Furthermore, immunostaining indicated that hESCs were positive for *OCT4*, whereas hESC-CMs stained positive for cardiac-specific markers *MYH6* (Fig. 1d), approving that these cells were successfully differentiated into cardiomyocytes.

### *MiR-335* expression pattern during the human cardiac differentiation process

**Fig. 2:**
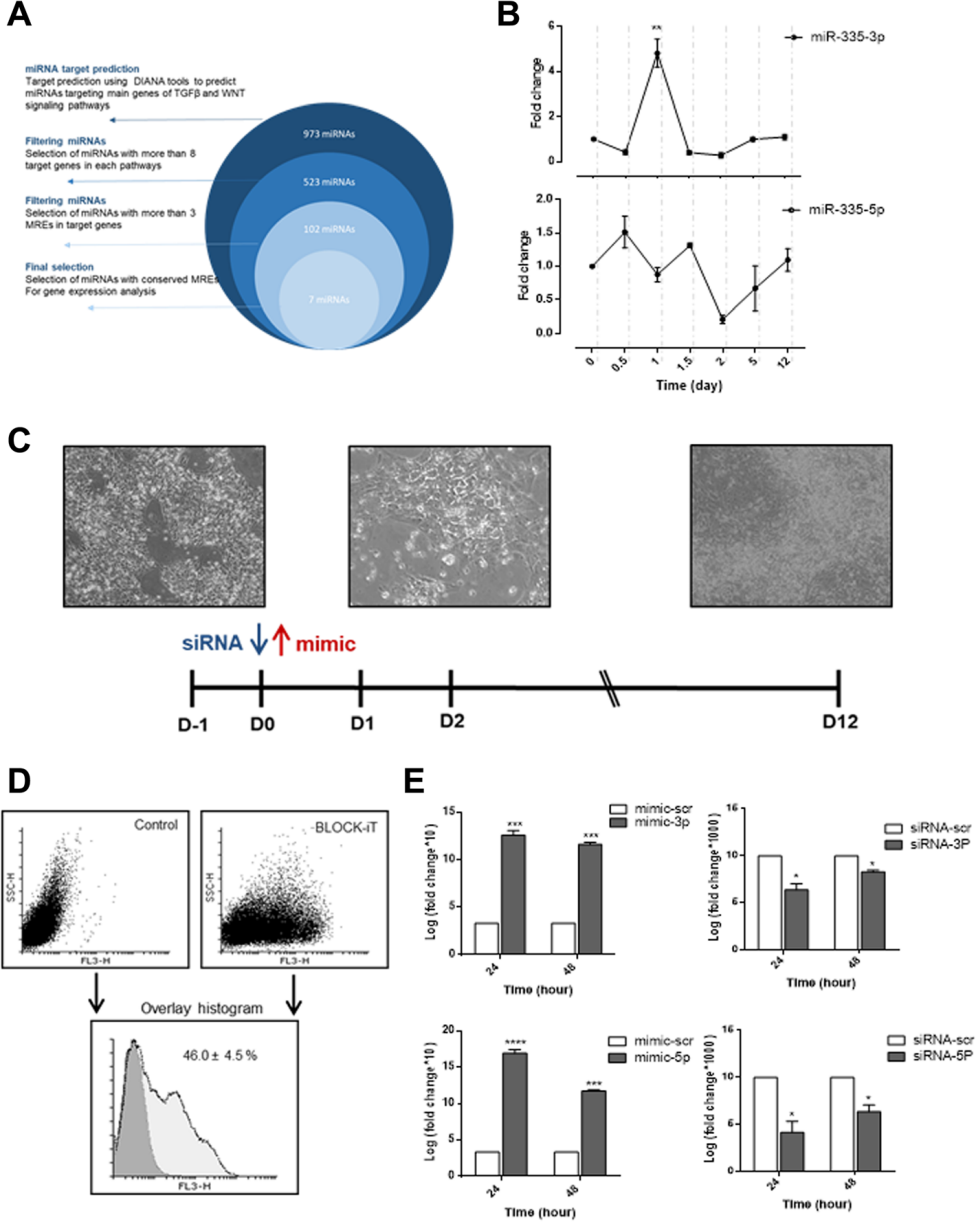
*MiR-335* expression alteration during cardiac differentiation. **a** Schematic presentation of different stages of miRNA screening. **b** *miR-335-3p* (top) and *miR-335-5p* (bottom) expression pattern during hESC cardiac differentiation. **c** Gain- and loss-of-function strategy in which *miR-335*-specific mimics and siRNAs were transfected on day 0 of differentiation process**. d** Flow cytometry results showed that hESCs were efficiently transfected by *miR-335* specific mimics or siRNAs along with 50 nM BLOCK-iT Alexa Fluor Red Fluorescent oligonucleotide. **e** The RT-qPCR confirmed significant (*P* value < 0.05) overexpression of *miR-335-3p* (top-left) and *miR-335-5p* (bottom-left) but significant downregulation of *miR-335-3p* (top- right) and *miR-335-5p* (bottom-right), 24 and 48 h after transfection. RT-qPCR data are presented as mean ± SEM normalized against day 0 data. *GAPDH* was used as a housekeeping gene

*MiR-335-3p* and *miR-335-5p* expression status was measured at seven time points of human cardiac differentiation process, using RT-qPCR. Both *miR-335-3p* and *miR-335-5p* had relatively high levels of expression during this process (data not shown); however, their expression pattern was different. While *miR-335-3p* was transiently upregulated at day 1 and substantially downregulated to the end of differentiation process, *miR-335-5p* showed no significant alteration during the differentiation (Fig. 2b).

### The effect of *MiR-335* expression alteration on cardiac differentiation

**Fig. 3:**
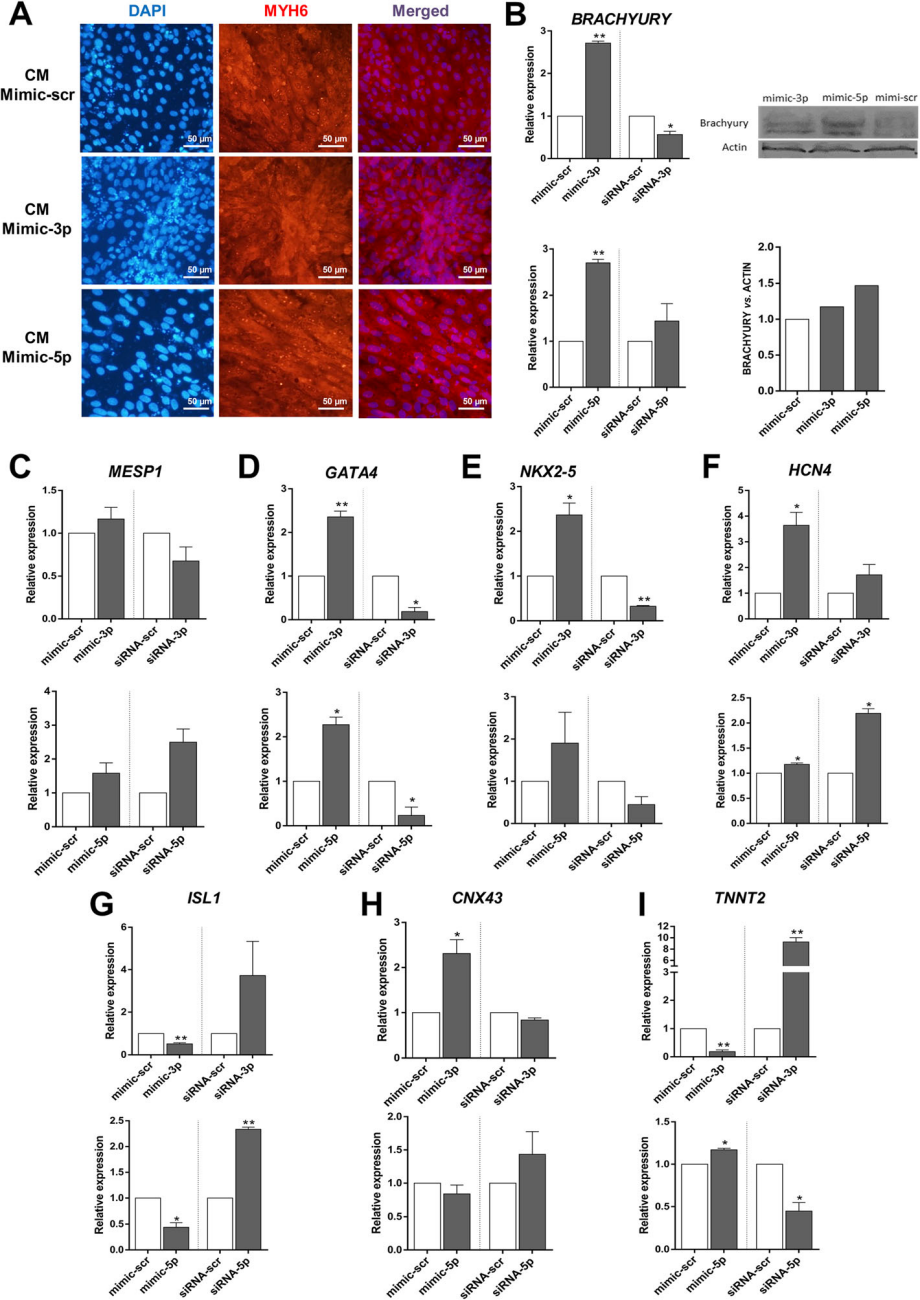
*MiR-335* regulates earlier stages of hESC cardiac differentiation. **a** Immunocytochemistry analysis showed that overexpression of *hsa-miR335-3p* or *hsa-miR335-5p* on day 0 did not affect the final outcome of cardiac differentiation on day 12 (cardiomyocytes), as detected by *MYH6* expression in all groups. **b** Overexpression of both *hsa-miR335-3p* (top) and *hsa-miR335-5p* (bottom) resulted in significant elevation of *BRACHYURY* (mesoderm marker) expression. Consistently, *hsa-miR335-3p* downregulation resulted in reduced expression of *BRACHYURY*. Western blot results indicate an increased level of BRACHYURY following the mimic-3p and mimic-5p treatment, compared to mimic-scr control (right). **c** No significant expression alteration was detected for *MESP1* following alterations in *hsa-miR335* expression. **d**, **e** Expressions of *GATA4* and *NKX2-5* (main cardiac progenitor markers) were both increased following *miR-335-3p* (top) and *miR-335-5p* (bottom) overexpression. Downregulation of *miR-335* had reverse effect on both of these cardiac progenitor markers. **f** Expression of *HCN4* (the first heart field marker) was significantly increased following *miR-335-3p* (top) and *miR-335-5p* (bottom) overexpression. **g** Alterations in the expression of *miR-335-3p* (top) and *miR-335-5p* (bottom) had a significant reverse effect on *ISL1* (the second heart field marker) expression. **h**, **i** *Has-miR-335-3p* (top) overexpression increased *CNX43* expression but decreased *TNNT2* expression. However, *has-miR-335-5p* decreased *CNX43* but enhanced *TNNT2* expressions*.* RT-qPCR data are presented as mean ± SEM normalized against mimic-scr and siRNA-scr. *GAPDH* was used as a housekeeping gene

In order to examine the effect of *miR-335-3p/5p* expression alteration on cardiac differentiation, gain- and loss-of-function study was performed according to *miR-335-3p* and *miR-335-5p* expression profiles (Fig. 2c). In this part, *miR-335-3p* and *miR-335-5p* mimics or their corresponding siRNAs were transfected into differentiating hESCs on D0 to induce specific overexpression and downregulation, respectively. Red fluorescent oligo transfection indicated ~ 45% transfection efficiency (Fig. 2d), and specific up- and downregulation of each miRNA was further confirmed using RT-qPCR, 24 and 48 h post transfection (Fig. 2e). Immunostaining of the cells transfected by mimic-scr, mimic-3p, or mimic-5p indicated successful cardiac differentiation (Fig. 3a). To determine the differentiation stage which has been most affected by *miR-335* expression alteration, the expression level of stage-specific markers was measured by RT-qPCR. Here, the expressions of *BRACHUYRY* and *MESP1* as mesodermal markers, *NKX2-5* and *GATA4* as cardiac progenitor markers, *HCN4* and *ISL1* as progenitor markers of the first and second heart field, and *TNNT*2 and *CNX43* as cardiomyocyte markers were analyzed. RT-qPCR data indicated that 48 h post-transfection of mimic-3p or mimic-5p, the expression level of *BRACHYURY* was significantly increased (Fig. 3b). Consistently, downregulation of *miR-335-3p* was followed by significant reduction of *BRACHUYRY* expression (Fig. 3b). The results of western blotting also confirmed the increased protein level of BRACHYURY following mimic-3p and mimic-5p treatments compared to the mimic-scr control (Fig. 3b, right). Increased or decreased level of *miR-335* did not show a significant effect on *MESP1* expression level (Fig. 3c). Interestingly, *GATA4* and *NKX2-5* expression levels were significantly increased following *miR-335-3p* overexpression while they decreased after using their related siRNA (Fig. 3d, e). Similar significant results were obtained for *miR-335-5p* against *GATA4*, but results were non-significant against *NKX2-5* expression (Fig. 3d, e).

Following transfection of mimic-3p and mimic-5p, *HCN4* (the first heart field marker) was significantly upregulated as well; however, *miR-335-3p* and *miR-335-5p* downregulation results were not consistent with it (Fig. 3f). *MiR-335* expression alteration also has effect on *ISL1* (the second heart field marker) expression. Transfection of both mimic-3p and mimic-5p resulted in downregulation of *ISL1* expression while, *miR-335* downregulation resulted in increased *ISL1* expression (Fig. 3g). Overexpression of *miR-335-3p* significantly upregulated *CNX43* expression but suppressed the expression of *TNNT2* at the final stage of the differentiation process (Fig. 3h, i). Interestingly, *miR-335-5p* overexpression could significantly upregulate *TNNT*2 with no significant effects on *CNX43* expression.

### *MiR-335* as an activator of WNT signaling pathway

**Fig. 4:**
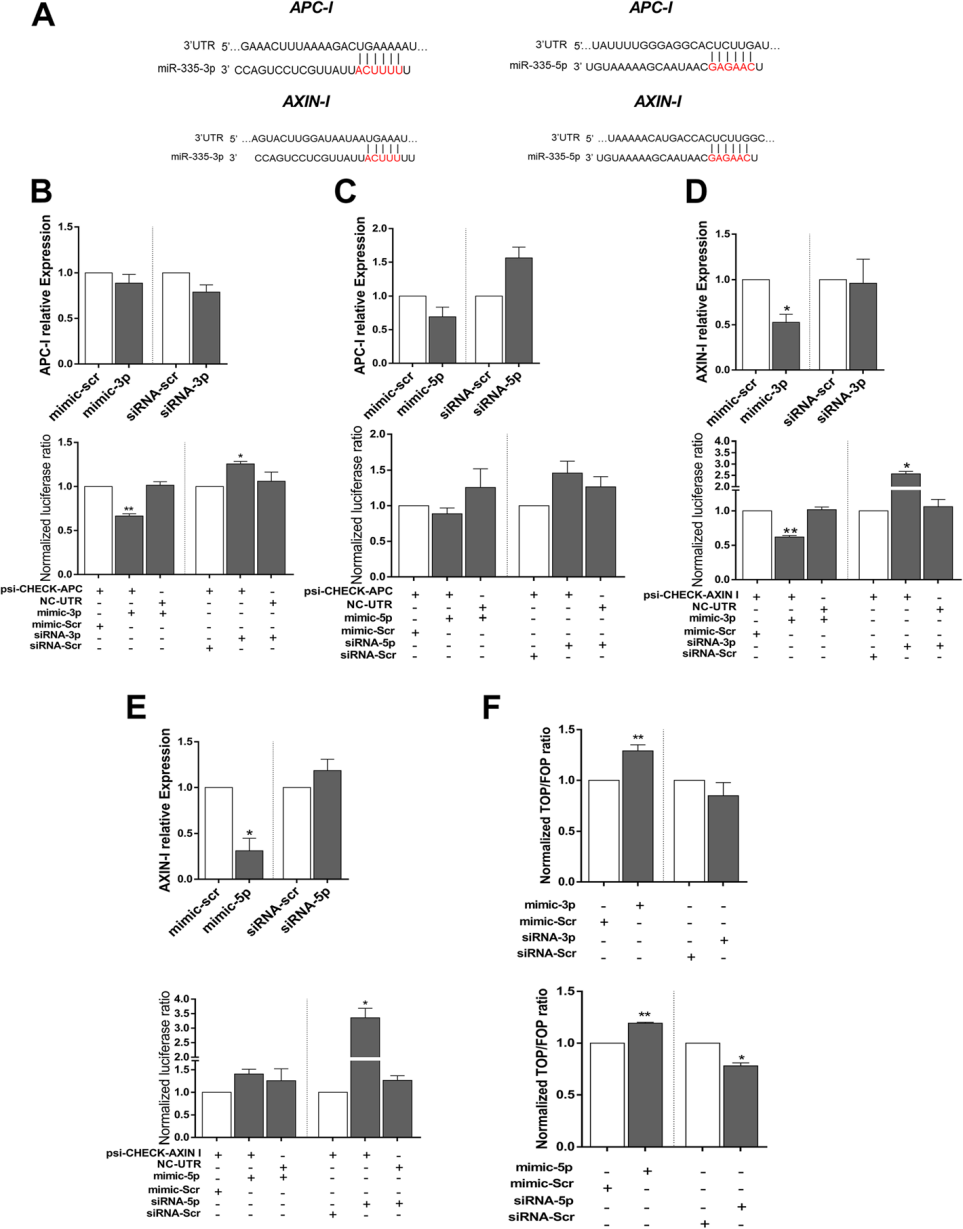
*MiR-335* activates WNT signaling through targeting *APC* and *AXIN-I*. **a** Pairing status of *miR-335-3p* and *miR-335-5p* with MREs within 3′UTR sequences of *APC* and *AXIN-I*. **b** Alterations in the expression of *miR-335-3p* did not significantly change the transcription level of *APC* gene (top); however, dual luciferase assay supported a direct interaction between them (bottom). **c** Neither RT-qPCR nor dual luciferase assay showed an interaction between *miR-335-5p* and *APC*. **d** Alterations in the expression of *miR-335-3p* significantly changed the *AXIN-I* transcript level (top), and dual luciferase assay indicated a direct interaction between them (bottom). **e** Although RT-qPCR suggested downregulation of *AXIN-I* following *miR-335-5p* overexpression (top), dual luciferase assay did not show an interaction between them (bottom). **f** Overall inductive effect of both *miR-335-3p* (top) and *miR-335-5p* (bottom) on WNT signaling as detected by TOP/FOP flash assay. All experiments were done in three biological replicates and results are presented as mean ± SEM

Target prediction tools predicted 5 and 1 potential target sites for *miR-335-3p* within the 3′UTR sequences of *APC* and *AXIN-I* genes, respectively. For *miR-335-5p*, a single recognition site was predicted within 3′UTR sequences of both *APC* and *AXIN-I* genes (Fig. 4a). Alterations in the expression of *APC* and *AXIN-I* target genes were investigated 48-h post-transfection of *miR-335* mimics or siRNAs on D0 of hESC differentiation. Also, direct interaction of each miRNA mimic and 3′UTR sequences of *APC* and *AXIN-I* predicted target genes was investigated in HEK293 cells, using dual luciferase reporter assay.

RT-qPCR indicated that up- or downregulation of both *miR-335-3p* (Fig. 4b, top) and *miR-335-5p* (Fig. 4c, top) did not significantly affect *APC* gene expression in differentiating cells. Co-transfection of reporter construct containing *APC*-3′UTR and *miR-335-3p* mimic in HEK293 cells resulted in a significant reduction of luciferase activity, and consistently, luciferase activity was increased after the application of siRNA against *miR-335-3p*, compared to scrambled controls (Fig. 4b, bottom). This effect was abrogated using off target UTR (the same length of UTR with no available target site for miRNA), thereby approving the direct interaction between *miR-335-3p* and *APC* 3′UTR. Similar dual luciferase assay results indicated that *miR-335-5p* was not interacting with APC 3′UTR sequence (Fig. 4c, bottom).

Transfection of differentiating hESCs with *miR-335-3p* mimic resulted in significant downregulation of *AXIN-I* expression, compared to scrambled control transfection (Fig. 4d, top). Furthermore, dual luciferase assay confirmed the direct interaction between *miR-335-3p* mimic and 3′UTR sequence of *AXIN-I* (Fig. 4d, bottom). Transfection with *miR-335-5p* mimic also caused a significant reduction in *AXIN-I* transcripts as examined by RT-qPCR (Fig. 4e, top). However, dual luciferase assay did not show a direct interaction between *miR-335-3p* and 3′UTR sequence of *AXIN-I* gene (Fig. 4e, bottom).

TOP/FOP flash assay indicated that overexpression of *miR-335-3p* or *miR-335-5p* led to significant upregulation of WNT signaling pathway in SW480 cells, compared to mimic-scr controls (Fig. 4f). Similar results to those of Top/Fop flash assay were observed when *miR-335* was downregulated by specific siRNA (Fig. 4f). The RT-qPCR analysis against *CYCLIND1* and c*-MYC* (downstream targets of WNT signaling pathway) also confirmed the upregulation of WNT signaling, following *miR-335-5p* overexpression (Additional file 1: Figure S1). Alterations in *miR-335-3p* expression did not significantly affect *CYCLIND1* and c*-MYC* expression (Additional file 1: Figure S1). Altogether, these data suggested that *miR-335-3p* might enhance the WNT signaling through targeting the inhibitory components of WNT signaling pathway (i.e., *APC* and *AXIN-I*) while *miR-335-5p* could activate this pathway indirectly without any direct effect on these two target genes.

### *MiR-335* as an inducer of TGFβ signaling pathway

**Fig. 5:**
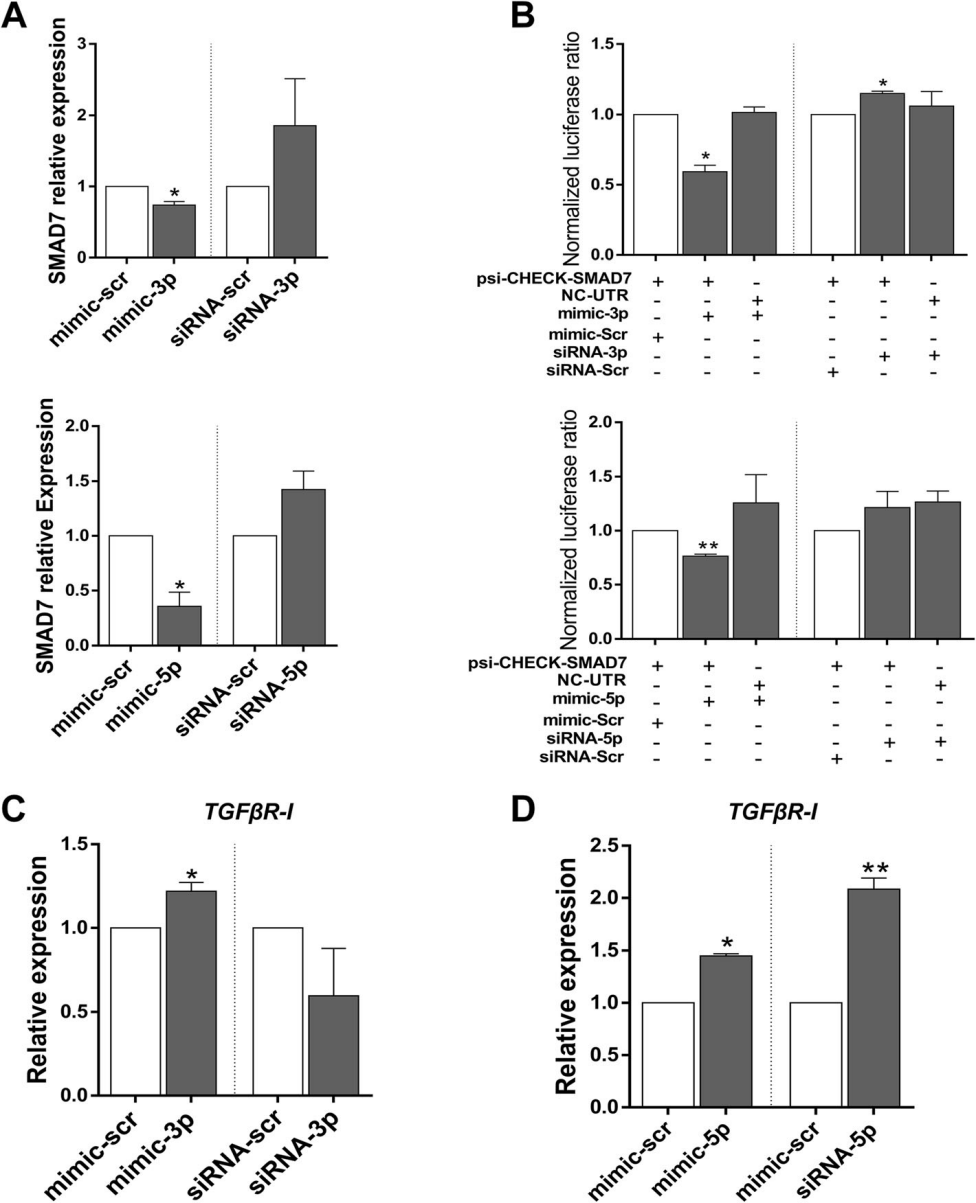
*MiR-335* activates TGFβ signaling pathway through targeting *SMAD7*. **a** Alterations in the expression of *SMAD7* followed by overexpression and downregulation of *miR-335-3p* (top) and *miR-335-5p* (bottom). **b** Dual luciferase assay study confirmed a direct interaction between *miR-335-3p* (top) and *miR-335-5p* (bottom), and *SMAD7* 3′UTR. Alteration in the expression of *TGFβR-I* was assessed by RT-qPCR followed by *miR-335-3p* and *miR-335-5p* overexpression (**c**) and downregulation (**d**) in hESC. RT-qPCR data are presented as mean ± SEM normalized against mimic-scr and siRNA-scr. GAPDH was used as a housekeeping gene

The in silico analyses suggested that *miR-335* could regulate TGFβ signaling through targeting *SMAD7* transcripts. It was predicted that both *miR-335-3p* and *miR-335-5p* target inhibitory *SMAD7* gene (Additional file 1: Figure S2A). RT-qPCR indicated that *SMAD7* expression was significantly reduced following *miR-335-3p* and *miR-335-5p* overexpression, 48 h after transfection in differentiating hESCs. Consistently, downregulation of these miRNAs had reverse effects (though non-significant) on *SMAD7* expression (Fig. 5a, top).

Luciferase activity was decreased following *miR-335-3p* overexpression, and this suppression was relieved by downregulation of *miR-335-3p* as detected by dual luciferase assay (Fig. 5b, top). Significant reductions in luciferase activity were also detected after *miR-335-5p* overexpression (Fig. 5a, bottom), while *miR-335-5p* downregulation could not rescue luciferase activity, significantly (Fig. 5b, bottom). Overall, these data suggested that both *miR-335-3p* and *miR-335-5p* were capable of targeting *SMAD7* transcript. The effect of *MiR-335* overexpression on other TGFβ signaling pathway components was also investigated using RT-qPCR. Results indicated that both *miR-335-3p* and *miR-335-5p* overexpression led to *TGFβR-I* upregulation (Fig. 5c, d). Alterations in the expression of *miR-335* had no significant effect on *SMAD2* and *SMAD3* expression (Additional file 1: Figure S2B-C). Altogether, these data suggest that both *miR-335-3p* and *miR-335-5p* might activate TGFβ signaling pathway through targeting *SMAD7* transcript.

## Discussion

TGFβ and WNT signaling pathways have some pleiotropic and multifunctional effects and regulate several biological processes including embryonic development, as well as cell differentiation, proliferation, and survival [25–27]. These two pathways play critical roles in orchestration of cardiac development and differentiation [28–30]. It is known that regulation of TGFβ and WNT signaling pathway is essential for mesoderm cell fate commitment, as well as cardiomyocyte progenitor cell formation and cardiomyocyte maturation [31]. These cardiogenesis steps are closely regulated by different mechanisms at posttranscriptional and posttranslational levels. MiRNAs are known to regulate different bioprocesses through targeting related signaling pathways [32–34]. For example, *hsa-miR-590-5p* was shown to be involved in cardiogenesis through regulating TGFβ signaling [35]. Also, *hsa-miR-23b* cluster was shown to induce proliferation in hepatocytes through TGFβ pathway inhibition [36] and *hsa*-*miR-126* is known to induce VEGF signaling and promote angiogenesis [37]. Here, we intended to introduce miRNAs which fine-tune TGFβ and WNT signaling pathways, during the cardiogenesis process. Bioinformatics analyses introduced 7 miRNAs which had multiple targets in both WNT and TGFB signaling pathways (Additional file 1: Table S2). Finally, *has-miR-335* that is well conserved in mammals and is under the control of mesoderm-specific enhancers in the second intron of *MEST* [38] (Additional file 1: Figure S3), was chosen for further investigation. Also, *miR-335* was reported to be involved in the fate commitment of mesoderm during the mouse embryonic differentiation [38]. *MiR-335-3p* (miRBase ID: MIMAT0004703) and *miR-335-5p* (miRBase ID: MIMAT0000765) were both highly expressed in mature heart [38]. It is predicted that these two miRNAs target different genes in WNT and TGFβ signaling pathways (Additional file 1: Tables S3–S4, Figure S4).

H5 human ESCs were successfully differentiated into beating cardiomyocytes after 12 days (Fig. 1a). Then, the expression pattern of mesoderm, cardiac progenitor, and mature cardiomyocyte molecular markers defined the span of each stage (Fig. 1b) which was further confirmed by flow cytometry (Fig. 1c) and ICC results (Fig. 1d). RT-qPCR results indicated a distinct expression pattern for *miR-335-3p* and *miR-335-5p* during the cardiac differentiation process. While *miR-335-3p* expression was significantly altered (up to six folds) during the mesoderm stage (before day 1.5), *miR-335-5p* was changed much less (1.5-fold) at this stage. *miR-335-5p* expression was strongly altered at the progenitor stage (after day 1.5) (Fig. 2b). Up- and downregulation of *miR-335* since D0 of the differentiation process lasted until the progenitor stage of the process, 2 days post-transfection (Fig. 2e). ICC results indicated that *miR-335* expression alterations did not change the fate of differentiating cells towards cardiomyocytes (Fig. 3a). RT-qPCR as well as western blot results indicated that both *miR-335-3p* and *miR-335-5p* gain of functions enhanced mesodermal cell commitment, as mirrored by the increased *BRACHYURY* expression level. Results of loss-of-function study also supported gain-of-function results for *miR-335-3p* (Fig. 3b)*.* It was consistent with a previous report showing that *miR-335* stabilizes the lineage decision in mouse ESCs for mesendoderm formation [38]. Also, Schoeftner et al. consistently reported that *miR-335* targets *OCT4* and *Rb* to control mESC proliferation and induced differentiation [39].

Following *miR-335-3p* or *miR-335-5p* gain- and loss-of-functions, cardiac progenitor markers (*GATA4* and *NKX2-5*) were also up- and down-regulated, respectively (Fig. 3d, e). Upregulation of *GATA4* and *NKX2-5* progenitor markers could enhance the first heart field progenitor cell commitment through upregulating *HCN4* (Fig. 3f) and downregulating *ISL1* expression (Fig. 3g). RT-qPCR results against late cardiac differentiation markers (*TNNT2* and *CNX43*) suggest that *miR-335-3p* and *miR-335-5p* might have complementary effects on the process.

While *miR-335-3p* downregulated *TNNT2* expression, *miR-335-5p* significantly upregulated it within the cells (Fig. 3i). Similar results were also obtained for *CNX43* expression after alterations in *miR-335-3p* and *miR-335-5p* expression (Fig. 3h). Altogether, these data suggest that *miR-335* enhances cardiac differentiation through upregulation of the expression of mesoderm (*BRACHYURY*) and cardiac progenitor marker (*GATA4* and *NKX2-5*) genes. Nevertheless, molecular mechanism(s) via which these miRNAs regulate the expression of these markers remained undiscovered.

Bioinformatics analyses indicated that *miR-335* regulates WNT and TGFβ signaling pathways. Moreover, RT-qPCR (Fig. 4b–e, top) and dual luciferase assay (Fig. 4b–e, bottom) showed that *miR-335-3p* specifically target 3′UTR sequences of *APC* and *AXIN-I*, which are two main members of WNT inhibitor complex. These data suggested that *miR-335-3p* but not *miR-335-5p* could regulate *APC* expression at the post-transcriptional level.

Although *miR-335-5p* had no direct interaction with 3′UTR of these two target genes, but RT-qPCR results indicated a significant downregulation of *AXIN-I* transcript (Fig. 4e, top). Thus, it could be concluded that *miR-335-5p* indirectly downregulated *AXIN-I* expression without interacting with its transcripts. Consistently, both of these miRNAs were capable of activating WNT signaling pathway as detected by Top/Fop flash assay (Fig. 4f). This is also consistent with a previous report which showed that *miR-335-5p* activates WNT signaling pathway through *DKK1* downregulation [40].

Interaction between both *miR-335-3p* and *miR-335-5p* and *SMAD7* 3′UTR was also confirmed by RT-qPCR (Fig. 5a) as well as dual luciferase assay (Fig. 5b). Results indicated that these two miRNAs could enhance *TGFβR-I* expression (Fig. 5c, d). There are two SMAD-binding elements within *TGFβR-I* promoter recognized by SMAD7, which inhibit *TGFβR-I* expression [41]. Downregulation of *SMAD7* by these two miRNAs could lessen the inhibitory effect of *SMAD7* and enhance *TGFβR-I* expression. Altogether, the results proposed that *miR-335-3p* and *miR-335-5p* could activate WNT and TGFβ signaling pathways.

LEF-1/β-catenin complex is reported to bind to the TCF binding site at the *BRACHYURY* promoter sequence and enhance its expression [42]. TGFβ1 also induces the expression of *BRACHYURY* in human carcinoma cells [43]. In other words, activation of WNT and TGFβ signaling pathways could enhance *BRACHYURY* expression, as also observed in the current experiment. Furthermore, WNT signaling pathway activation was shown to enhance *GATA4* [44] as well as *NKX2-5* expression through hindering *HDAC1* inhibitory effect [45]. There is also evidence showing that TGFβ activation enhances *GATA4* and *NKX2-5* expression [46–48].

## Conclusion

**Fig. 6:**
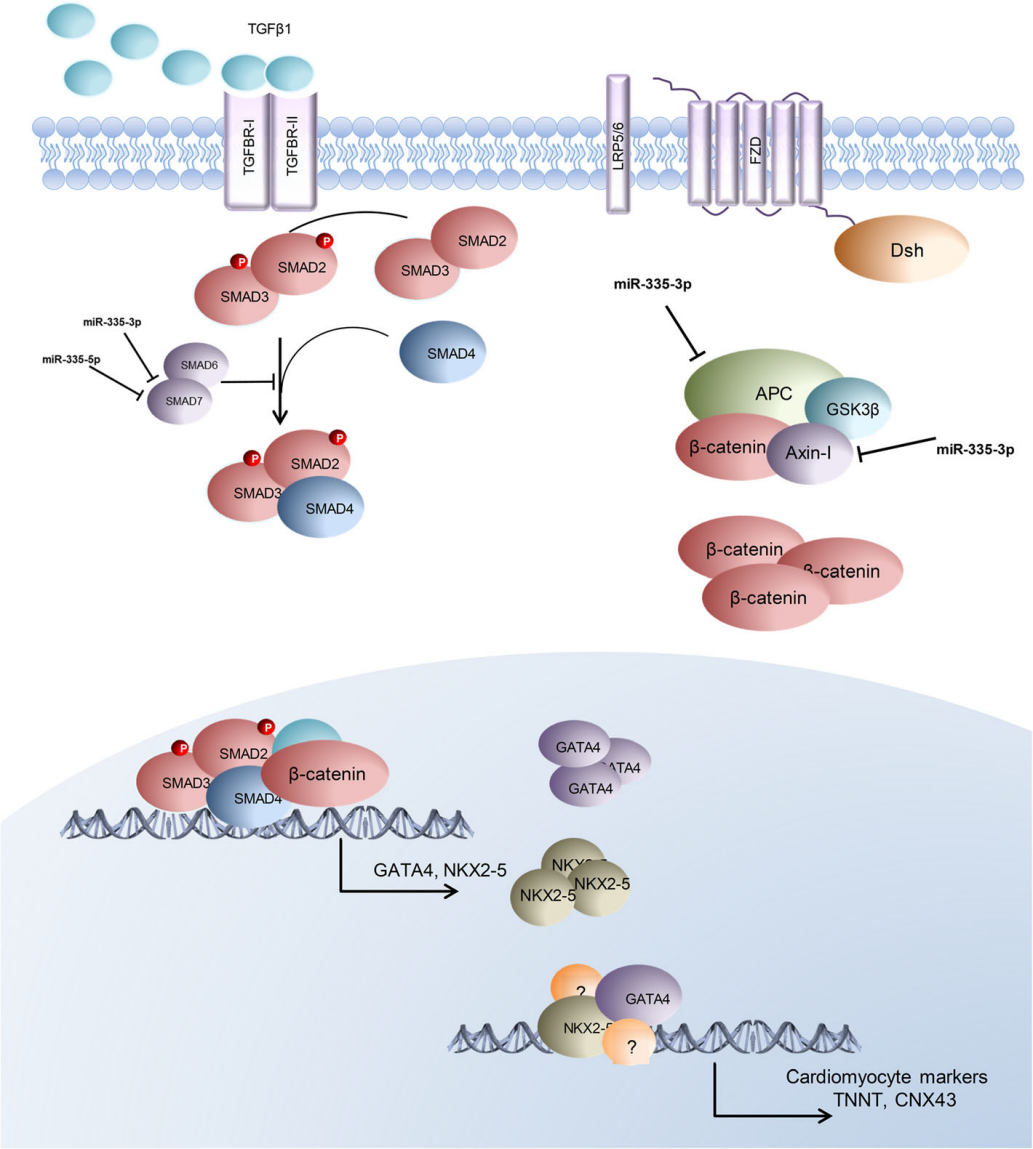
Schematic presentation of *miR-335* involvement in cardiomyocyte differentiation procedure (details are discussed in the text)

Based on the bioinformatics and experimental validations, we propose a regulatory network for *miR-335-3p* and *miR-335-5p* during cardiomyocyte differentiation (Fig. 6). Accordingly, both *miR-335-3p* and *miR-335-5p* might activate WNT and TGFβ signaling pathways that, in turn, induce mesoderm (*BRACHYURY*) and progenitor (*GATA4* and *NKX2-5*) marker expression and cardiac differentiation.

## Additional files

Additional file 1: **Table** **S1.** Primer sequences used in this research. **Table S2**. Final list of candidate miRNAs. **Table S3**. List of potential miR-335-3p target genes related to WNT and TGFβ signaling pathways. **Table S4**. List of potential miR-335-5p target genes related to WNT and TGFβ signaling pathways. **Figure S1**. RT-qPCR results of *C-MYC* and *CCND1* after mimics and siRNA treatment for miR-335-3p (A) and miR-335-5p (B). All experiments were done in three biological replicates and presented as mean ± SEM. **Figure S2**. RT-qPCR results of *SMAD2* and *SMAD3* expression*.* A) Pairing status of *miR-335-3p* (left) and *miR-335-5p* (right), with 3′UTR of *SMAD7* gene. B) *SMAD2* expression was not significantly changed followed by *miR-335-3p* (top) and *miR-335-5p* (bottom) overexpression. C) RT-qPCR data also showed no significant alterations in *SMAD3* expression following *miR-335-3p* (top) and *miR-335-5p* (bottom) overexpression. All data are presented as mean ± SEM normalized against mimic-scr and siRNA-scr. GAPDH was used as a housekeeping gene. **Figure S3**. Genomic location of *mir-335* presented in UCSC genome browser. *miR-335* is located within the second intron of *MEST* gene, containing two conserved mature miRNAs (highlighted in red) including *miR-335-3p* and *miR-335-5p*. **Figure S4**. The potential targets of *miR-335* in TGFβ (A) and WNT (B) signaling pathways according to the KEGG pathway. The target genes are marked with red stars. (DOCX 474 kb)

Additional file 2: **Movie S1.** (AVI 2586 kb)
