# Supplementary material for: Hsa-miR-335 regulates cardiac mesoderm and progenitor cell differentiation
Source: Stem Cell Res Ther. 2019 Jun 27;10:191. doi: 10.1186/s13287-019-1249-2 (PMC6595595; doi:10.1186/s13287-019-1249-2)
Supplement: Supplementary file 1 — Table S1. Primer sequences used in this research. Table S2. Final list of candidate miRNAs. Table S3. List of potential miR-335-3p target genes related to WNT and TGFβ signaling pathways. Table S4. List of potential miR-335-5p target genes related to WNT and TGFβ signaling pathways. Figure S1. RT-qPCR results of C-MYC and CCND1 after mimics and siRNA treatment for miR-335-3p (A) and miR-335-5p (B). All experiments were done in three biological replicates and presented as mean ± SEM. Figure S2. RT-qPCR results of SMAD2 and SMAD3 expression. A) Pairing status of miR-335-3p (left) and miR-335-5p (right), with 3′UTR of SMAD7 gene. B) SMAD2 expression was not significantly changed followed by miR-335-3p (top) and miR-335-5p (bottom) overexpression. C) RT-qPCR data also showed no significant alterations in SMAD3 expression following miR-335-3p (top) and miR-335-5p (bottom) overexpression. All data are presented as mean ± SEM normalized against mimic-scr and siRNA-scr. GAPDH was used as a housekeeping gene. Figure S3. Genomic location of mir-335 presented in UCSC genome browser. miR-335 is located within the second intron of MEST gene, containing two conserved mature miRNAs (highlighted in red) including miR-335-3p and miR-335-5p. Figure S4. The potential targets of miR-335 in TGFβ (A) and WNT (B) signaling pathways according to the KEGG pathway. The target genes are marked with red stars. (DOCX 474 kb) [file 13287_2019_1249_MOESM1_ESM.docx]

***Hsa-miR-335* regulates cardiac mesoderm and progenitor cell differentiation**

Maryam Kay^a^, Bahram Mohammad Soltani^a^*, Fahimeh Hosseini Aghdaei^a^, Hassan Ansari^b^, Hossein Baharvand^b,c*^

a: Department of Genetics, Faculty of Biological Sciences, Tarbiat Modares University, Tehran, Iran

b: Department of Stem Cells and Developmental Biology, Cell Science Research Center, Royan Institute for Stem Cell Biology and Technology, ACECR, Tehran, Iran

c. Department of Developmental Biology, University of Science and Culture, Tehran, Iran

Email: [Maryam_kay2001@yahoo.com](mailto:Maryam_kay2001@yahoo.com), [hoseiny6965@gmail.com](mailto:hoseiny6965@gmail.com), [hassanansaritat@yahoo.com](mailto:hassanansaritat@yahoo.com)

*Correspondences:

Bahram M. Soltani, Department of Molecular Genetics, Faculty of Biological Sciences, Tarbiat Modares University, Tehran, Iran, 111-14115, Phone: +98-21-82884703, Fax: +98-21-82883463, Email: [soltanib@modares.ac.ir](mailto:soltanib@modares.ac.ir)

Hossein Baharvand, Royan Institute, Banihashem Sq., Banihashem St., Ressalat highway, Tehran, Iran. Postal Code: 1665659911, P.O. Box: 16635-148, Tel: +98 21 22306485, Fax: +98 21 23562507 ; E-Mail: [Baharvand@RoyanInstitute.org](mailto:Baharvand@RoyanInstitute.org)

A

B

**Figure S1)** RT-qPCR results of *C-MYC* and *CCND1* after mimics and siRNA treatment for miR-335-3p (A) and miR-335-5p (B). All experiments were done in three biological replicates and presented as mean ± SEM.


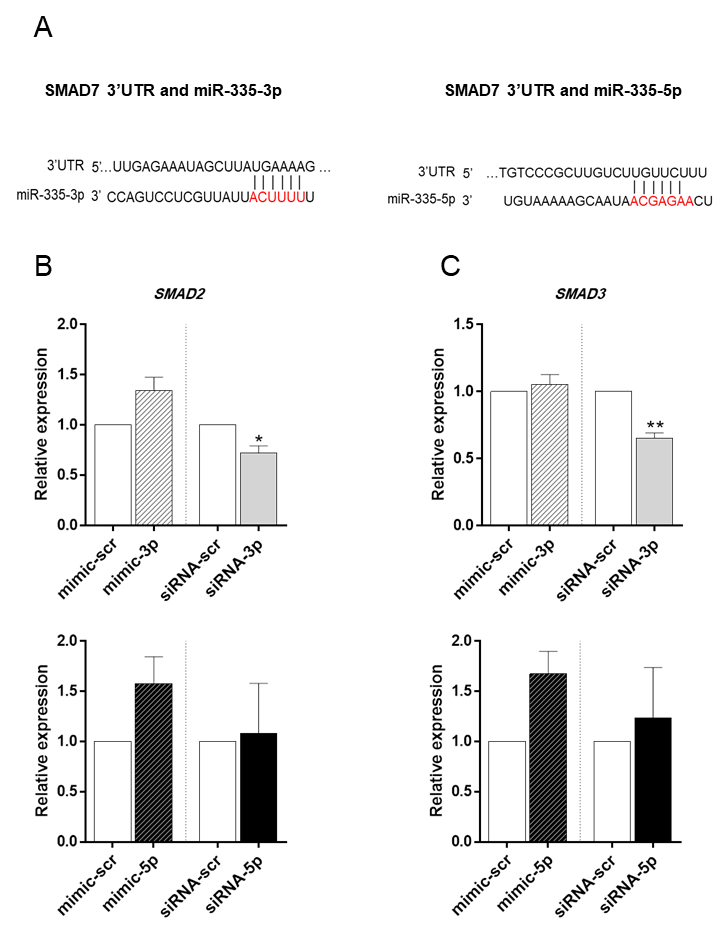


**Figure S2) RT-qPCR results of *SMAD2 and SMAD3* expression*.*** A) Pairing status of *miR-335-3p* (left) and *miR-335-5p* (right), with 3'UTR of *SMAD7* gene. B) *SMAD2* expression was not significantly changed followed by *miR-335-3p* (TOP) and *miR-335-5p* (bottom) overexpression. C) RT-qPCR data also showed no significant alterations in *SMAD3* expression following *miR-335-3p* (TOP) and *miR-335-5p* (bottom) overexpression. All data are presented as mean ± SEM normalized against mimic-scr and siRNA-scr. GAPDH was used as a housekeeping gene.


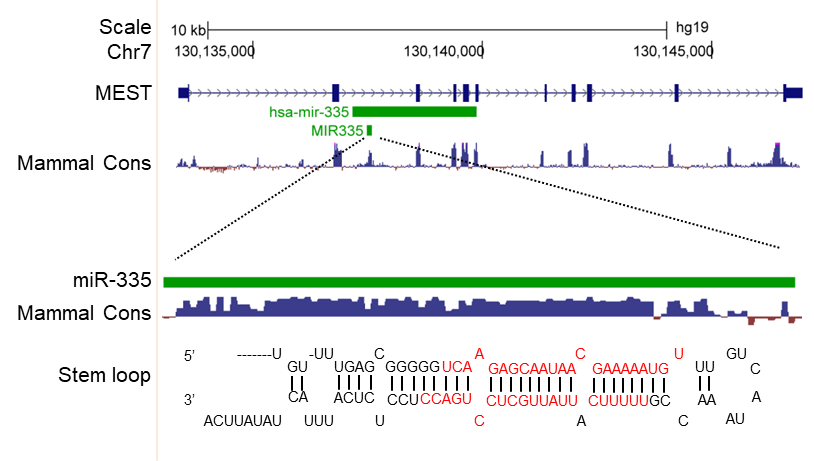


**Figure S3) Genomic location of *mir-335* presented in UCSC genome bowser.** *miR-335* is located within the second intron of *MEST* gene, containing two conserved mature miRNAs (highlighted in red) including *miR-335-3p* and *miR-335-5p*.


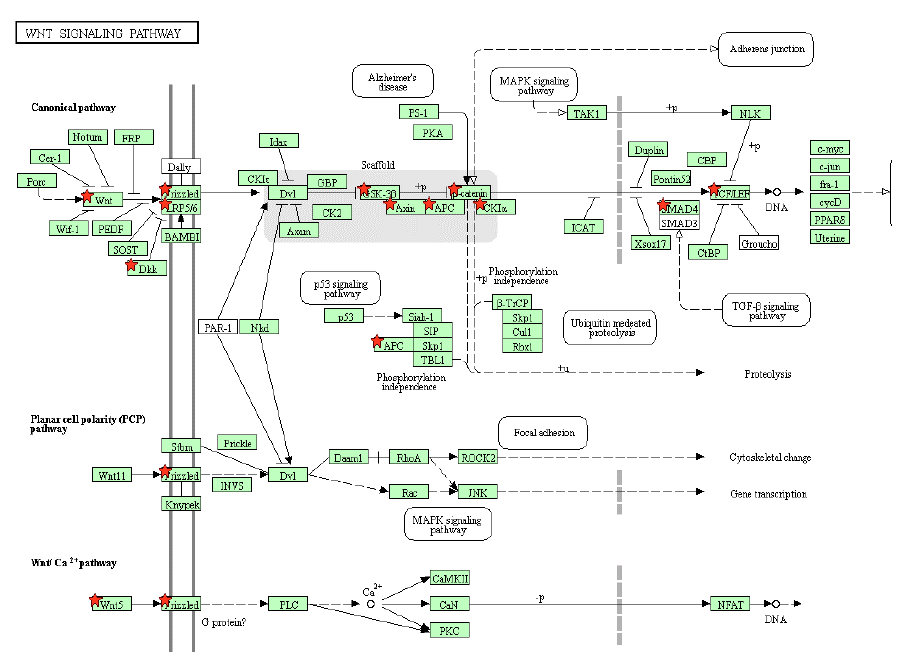

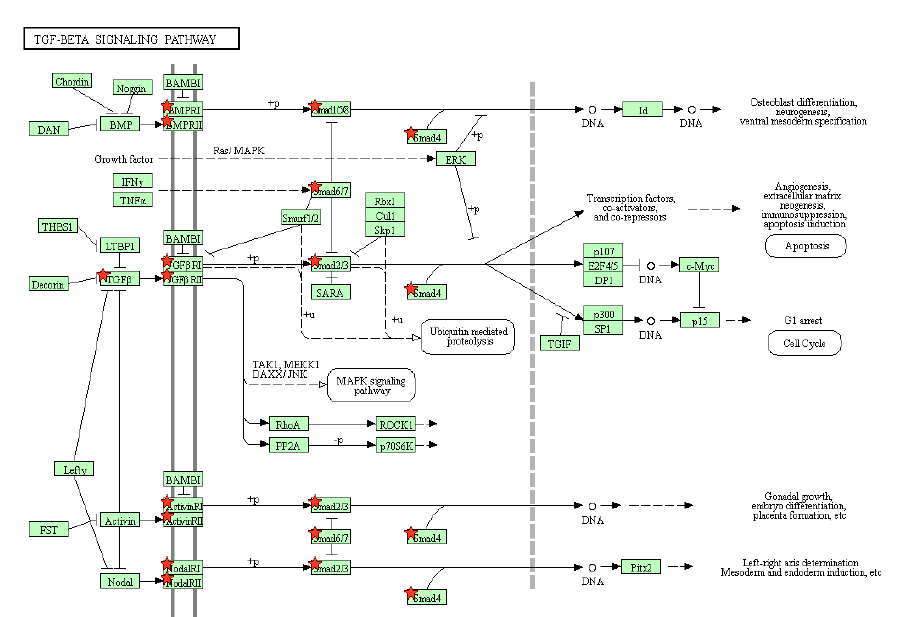


A

B

**Figure S4)** **The potential targets of *miR-335* in TGFβ (A) and WNT (B) signaling pathways according to the KEGG pathway.** The target genes are marked with red stars.

Table S1) Primer sequences used in this research.

| Primer name | **sequence** |
| --- | --- |
| *NANOG* | Forward: AAAGAATCTTCACCTATGCC  Reverse: GAAGGAAGAGGAGAGACAGT |
| *BRACHYURY* | Forward: AATTGGTCCAGCCTTGGAAT  Reverse: CGTTGCTCACAGACCACA |
| *MESP1* | Forward: ACCTTCGAAGTGGTTCCTTG  Reverse: TCCTGCTTGCCTCAAAGTGT |
| *GATA4* | Forward: CAGCAGCGAGGAGATGCGTC  Reverse: GCTGGTCTGTGGAGACTGGC |
| *NKX2-5* | Forward: TCTATCCACGTGCCTACAG  Reverse: CCTCTGTCTTCTCCAGCTC |
| *ISL1* | Forward: TACAAAGTTACCAGCCACC  Reverse: GGAAGTTGAGAGGACATTGA |
| *HCN4* | Forward: GAACAGGAGAGGGTCAAGTCG  Reverse: CATTGAAGACAATCCAGGGTGT |
| *CNX43* | Forward: GCTATGACAAGTCTTTCCCA  Reverse: CAGTTTCTCTTCCTTTCGCA |
| *TNNT2* | Forward: ATGATGCATTTTGGGGGTTA  Reverse: CAGCACCTTCCTCCTCTCAG |
| *MYH6* | Forward: ATTGCTGAAACCGAGAATGG  Reverse: CGCTCCTTGAGGTTGAAAAG |
| *TGFβR-I* | Forward: CATTTTTCCCAAGTGCCAGT  Reverse: ACACCCCTAAGCATGTGGAG |
| *TGFβR-II* | Forward: TTTGGATGGTGGAAGGTCTC  Reverse: GCAACAGCTATTGGGATGGT |
| *SMAD2* | Forward: CGAGTGGGTAGTGTTCTCAGGG  Reverse: TGGTCAGCTCCTTCTGGTGTG |
| *SMAD3* | Forward: CTTCCTAAGAGTCAAAGTCCCTGC  Reverse: CCTGTGCTGGAACATCATCTCAG |
| *SMAD4* | Forward: AAGTAATGGCTCTGGGTTGGG  Reverse: TCAAACAGCAGAACAAAGATAAGGAA |
| *SMAD7* | Forward: TGTCCAGATGCTGTGCCTTCCT  Reverse: CTCGTCTTCTCCTCCCAGTATG |
| *APC* | Forward: TATTACGGAATGTGTCCAGCTTG  Reverse: CCACATGCATTACTGACTATTGTC |
| *AXIN-I* | Forward: ATGCAGGAGAGCGTGCAGGTC  Reverse: TGACGATGGATCGCCGTCCTC |
| *C-MYC* | Forward: CTCCTACGTTGCGGTCACAC  Reverse: CGGGTCGCAGATGAAACTCT |
| *CCND1* | Forward: CAGAGTGATCAAGTGTGACCC  Reverse: CGTCGGTGGGTGTGCAAGC |
| *GAPDH* | Forward: GCCACATCGCTCAGACAC  Reverse: GGCAACAATATCCACTTTACCAG |
| *Hsa-miR-335-3p* | Forward: GGGGGTTTTTCATTATTGCTCCTG |
| *Hsa-miR-335-5p* | Forward: GGGGTCAAGAGCAATAACGAAAAA |
| *Universal* | Reverse: AACTCAAGGTTCTTCCAGTCACG |

**Table S2) Final list of candidate miRNAs**

| miRNA | miRBase ID |
| --- | --- |
| Hsa-miR-335-3p | MIMAT0004703 |
| Hsa-miR-335-5p | MIMAT0000765 |
| Hsa-miR-3613-3p | MIMAT0017991 |
| Hsa-miR-140-3p | MIMAT0004597 |
| Hsa-miR-186 | MIMAT0004612 |
| Hsa-miR-5590 | MIMAT0022300 |
| Hsa-miR-5582-3p | MIMAT0022280 |
| Hsa-miR-3148 | MIMAT0015021 |

**Table S3) List of potential miR-335-3p target genes related to WNT and TGFβ signaling pathways.**

| Gene | miR-335-3P/WNT | | |
| --- | --- | --- | --- |
|  | **D/C** | **T/C** | **O/C** |
| *WNT5A* | 5/2+3- | 3/1+2- | 4/2+2- |
| *WNT3* | 2/1+1- | 2/2- | 3/3- |
| *WNT16* | 6/3+3- | 1/1- | 3/1-2+ |
| *FZD4* | 6/2+4- | 2/1+1- | 5/3+2- |
| *APC* | 12/12+ | 5/3+2- | 2/1+1- |
| *GSK3B* | 6/6+ | 1/1+ | - |
| *AXIN-1* | - | 1/1+ | 1/1+ |
| *DKK-1* | 2/2- | 2/2- | 2/2- |
| Gene | **miR-335-3p/TGFβ** | | |
| *BMPR1B* | 6/5+1- | 3/2+1- | 1/1- |
| *BMPR1A* | 3/3+ | 3/2+1- | 1/1+ |
| *SMAD4* | 4/2+2- | 3/2+1- | 6/3+3- |
| *SMAD2* | - | 3/2+1- | 6/2-4+ |
| *SMAD7* | 2+ | 2/2+ | 2/2+ |

D: DIANA tools, T: Target Scan, O: Other tools including miRMap, StarMir, etc., and C: Conservation status of each MRE (miRNA recognition element). + indicates highly conserved while – marks poorly conserved MREs.

| Gene | miR-335-5P/WNT | | |
| --- | --- | --- | --- |
|  | **D/C** | **T/C** | **O/C** |
| *WNT10A* | - | 1/1+ | 2/2- |
| *FZD4* | - | - | 2/2- |
| *APC2* | - | - | 2/1+1- |
| *APC* | - | 1/1+ | 1/1+ |
| *AXIN-1* | - | - | 1/1+ |
| *AXIN-2* | - | 1/1- | 2/1+1- |
| Gene | **miR-335-5p/TGFβ** | | |
| *TGFB2* | - | 1/1+ | 1/1+ |
| *BMPR2* | - | - | 4/2+2- |
| *BMPR1B* | 4/1+3- | - | 3/3- |
| *ACVR2A* | - | 1/1+ | 2/2+ |
| *SMAD4* | - | 1/1+ | 1/1- |
| *SMAD2* | - | - | 4/1+3- |
| *SMAD5* | - | 1/1+ | 2/2- |

**Table S4) List of potential miR-335-5p target genes related to WNT and TGFβ signaling pathways.**

D: DIANA tools, T: Target Scan, O: Other tools including miRMap, StarMir, etc., and C: Conservation status of each MRE (miRNA recognition element). + indicates highly conserved while – marks poorly conserved MREs.
